# Chicago Medical Society

**Published:** 1887-12

**Authors:** 


### CHICAGO MEDICAL SOCIETY.

STATED MEETING, OCTOBER 27, 1887.

The President, W. T. Belfield, M. D., in the Chair.

Professor E. Fletcher Ingals reported a case of

RETROPHARYNGEAL ABSCESS

of unusual interest, on account of the deep fistulous tract which remains, and as an illustration of the necessity for prompt incision for the evacuation of pus in cases of retropharyngeal abscess.

Mrs. H. P., æt. 44, was sent to him during the month of August from a neighboring state for an opinion. She complained of an oppressive, purulent discharge from the pharynx, with more or less pain between the shoulders, cough and night sweats. She stated that the discharge would occur every few days, and that afterwards she would be free from cough, pain or other annoyance for some time, when the symptoms would again come on gradually, and increase steadily until another evacuation of the pus occurred.

Six months before coming to see him, she had had soreness and swelling in the naso-pharynx. The swelling had gradually extended downward, causing more or less difficulty in swallowing and respiration, and had been allowed to take its course for two months, when an opening was made in the lower part of the pharynx, which gave vent to a large quantity of offensive pus. Subsequently the incision closed, was reopened twice, and finally it had remained patent. While the swelling was still in the naso-pharynx, she had for some time also a purulent discharge from the right ear. He found the patient anæmic, weak and emaciated, weight only 94 pounds, instead of 112 pounds, as when in health. Pulse 132. Her tongue was red and glazed in center, but her appetite was good. The cough was not constant, but it usually troubled her considerably every day. The pains of which she complained were not severe, and were confined to the chest and shoulders.

Upon examination of the chest no abnormal signs were found, excepting slight feebleness of the respiratory sounds over the lower third of the right lung. A mucous polyp half closed the right naris ; the larynx was normal. When the tongue was forcibly depressed a little to the right of the median line, at the lower third of the larynx, a small red fungoid mass, about the size of a pea, could be seen. The vault of the pharynx was normal, and nothing else of an abnormal character could be seen. The fungoid mass was removed with a snare, and beneath it was found an opening about two millimeters in diameter, into which was passed a small elastic catheter, and gently pushed down a fistulous tract a distance of thirty centimeters, when she complained of its extremity causing her pain a little to the right of and below the right breast.

The position of the lower end of the fistula seemed in some way to account for the enfeebled respiration at the lower third of the right lung. The patient did not remain in the city, he, therefore, was unable to see her when there was pus in the cavity, and consequently could not determine the exact position of the lower end of the fistula.

He had recommended removal of the polypus and washing of the fistulous tract daily with carbolized water or peroxide of hydrogen through a catheter introduced to its bottom. The use of tonics was also advised. The result of the examination was communicated to her physician, but nothing further was heard of the case. Professor Ingals was under the impression that when the lower part of the sac was filled with pus it might be possible to detect it, and make a counter opening through the pleural sac, whereby constant drainage could be established so that healing of the fistula might be effected.

Professor F. O. Stockton said : "This sequel of a retropharyngeal abscess is something a little out of the ordinary run. In his experience he had never met with anything that produced any such symptoms, although he had frequently met with fistulas resulting from a retropharyngeal abscess. The case was interesting as showing what may occur when an abscess of this kind is not recognized in time. Abscesses are liable to burrow deeply, and in a location like this, where the soft tissues overlie a hard, bony substance, an abscess with a tendency to burrow could easily go very deep, and by pressing upon the nerves produce these pains; but it seems strange, unless the nerves were bare, that passing a small catheter down should produce these pains below the right breast. It looks as though some of the spinal nerves might have been laid bare by this burrowing of the pus. In a case of that kind, it seemed probable that what Professor Ingals had suggested was about the only thing that could be done, although one can hardly see how the opening he suggested could be made so as to give thorough drainage without injury to some nerve or other important structure.

Professor W. E. Casselberry said that the subject is one of interest for two reasons : *first*, retropharyngeal abscess is not a common disease, especially in the adult. It is oftentimes overlooked, and the responsibility rests upon the physician in all cases where there is dyspnœa, continuous or paroxysmal, to make a thorough examination of the throat both by inspection and palpation, in order to determine the diagnosis. *Secondly*, it is of interest on account of the peculiar complication in this case. He had seen the record of a similar case reported by Cohen among a number of cases of retropharyngeal abscess having some peculiarities. In Cohen's case a sinus burrowed deeply between the œsophagus and trachea, and finally pointed externally low down in the neck, and pus was evacuated by incision. On account of the loose connective tissue in this region and the influence of gravity, the natural tendency is for the pus to burrow downward, and other cases of the sort will doubtless be observed.

In reply to Dr. Strong, as to how he would make the opening suggested, Professor Ingals said he thought that when that sinus was filled with pus there might be a considerable collection--a pocket, perhaps, of a couple of inches in diameter—and if it was of that size, one could find the location and when found, he believed one could get an opening into it. He would not hesitate to plunge an aspirating needle into it, even passing it through the lung, if necessary. He was reminded of a case of an infant that he saw many years ago. He then thought the child had died of convulsions, but he thinks now that it died of retropharyngeal abscess. In young children sudden stopping of the breath occurs that seems as if from closure of the glottis, but there is none of the sound that occurs with spasm of the vocal chords. Recently a man of perhaps 50 years of age had been sent to him with a statement that he had stenosis of the larynx, and that something must be done. With a laryngeal mirror he examined his larynx, and found a large swelling in the pharynx, which one could see by depressing the tongue. It was a retropharyngeal abscess, which had existed four weeks. He swallowed with difficulty, but opening of the abscess relieved the dysphagia.

Albert B. Strong, M. D., reported two cases of

SUBPERIOSTEAL RESECTION OF A RIB FOR EMPYEMA IN CHILDREN; WITH RECOVERY.

He had within the past two years made subperiosteal resection of a segment of a rib for empyema in two young persons, one a boy of 6 and the other a youth of 17 years of age. The results had been so satisfactory that he desired to place the cases on record, particularly so since able authority has recently condemned the operation in children*.

Case I. In May, 1886, he had seen in consultation with Dr. Charles Venn, of this city, a boy 6 years of age. The father, mother and other children of the family were pictures of perfect health. The boy was large for his age, and up to the time of that illness had always enjoyed the best of health. Four weeks prior he began to cough, and eight or nine days later evidences of an effusion into the right pleural cavity were manifest. In spite of appropriate constitutional and local treatment, the effusion increased, and when Dr. Strong saw him the chest cavity was completely filled. The boy was much emaciated with continued fever and night-sweats.

A hypodermic needle thrust into the chest just above the eighth rib in the axillary line revealed the presence of pus. Under ether the eighth rib was exposed through a parallel incision two inches in length. The periosteum, midway between the upper and lower border of the rib, was split in a longitudinal direction for the distance of an inch. By means of a blunt hook the periosteum and intercostal vessels were

*See an article by Dr. Garnett, of Washington, on "The Surgical Treatment of Suppurative Pleuritis in Children," published in *The Journal of the American Medical Association*, September 17th, 1887.

easily peeled off from the posterior surface of the rib, and three-fourths of an inch was cut away with bone nippers. This was done without wounding the intercostal vessels or pleura. Next, the internal layer of the periosteum was split to the same extent, and parallel to the external layer. The pleura was opened, when two quarts of laudable pus was evacuated. The chest cavity was immediately washed out with a warm saturated solution of boracic acid, and a large rubber drainage tube was introduced and retained in position.

The subsequent history was uneventful. He began to improve at once. The chest cavity was daily washed out with simple warm water. A wad of oakum was placed over the wound. The tube was kept in place six weeks, when the lung entirely expanded and the wound completely healed. Sixteen months after the operation, the boy is perfectly well. The right chest is normal like its fellow, in shape and function. There is but a small scar at the seat of the operation, and the rib has perfectly reformed in direction and shape.

Case II.—A Norwegian boy, 17 years of age, always delicate, very thin and poorly nourished; six feet tall, pale and anæmic. His father, brother and one sister died of consumption. His mother is alive and doing well. The patient was first taken sick about March 10, 1887. The symptoms and signs indicated pleural pneumonia of the lower lobe of the right lung. In a few days he was convalescent.

On March 24 he had a relapse, and the right side of his chest slowly and intermittingly filled with effusion. He had night-sweats, diarrhœa and cough, and a temperature of 102° to 104° F. On April 24, about five weeks from the commencement of the effusion, empyema being diagnosed by the hypodermic syringe, with the assistance of Dr. Venn he removed, sub-periosteal, one and one-half inches of the ninth rib in the axillary line. Four quarts of laudable pus escaped. At the last about four ounces of clear serum passed out. The chest cavity was immediately washed out with a solution of boracic acid, and two large parallel rubber tubes with a drainage diameter of three-eighths of an inch each were introduced. These were held in position by a rubber shield and elastic bandage around the chest. The pleura was felt to be about three-fourths of an inch thick. During the next few days there were washed out large masses of lymph. Hot-water injections of two quarts at a time were used, night and morning. A wad of oakum was kept over the opening and changed often. The diarrhœa, sweating and cough began immediately to abate. The temperature in three days dropped to normal. On the first day of May, eight days after the operation, the tubes were crowded out. All the unfavorable symptoms had disappeared; he was eating and sleeping well. The opening on the inside was closed, as was then thought, by the expansion of the lungs; subsequently it was learned it was closed by the arching of the diaphragm. The tube was left out five days, when the temperature began to ascend, and soon reached 104° F. There was a return of the sweating, diarrhœa and cough. Nothing had escaped from the wound in the meantime. The wound was then reopened, and about five ounces of stinking pus and débris washed out. A large Van Buren urethral sound was passed into the chest, and it was ascertained that the lung was compressed into a small wad against the vertebral column. It had not expanded at all. He felt the apex of the sound in the chest above the clavicle. He then introduced longer, fenestrated drainage-tubes, three inches in length. From that time on his convalescence was uninterrupted. His temperature quickly became normal, and did not again rise. He soon began to go out of doors, and improved rapidly. On June 12, three weeks after the tubes had been introduced again, notes of the case read: Boy has gained ten pounds; out of doors most all day. Eats enormously and digests well. No cough, and but little discharge. The sound in the chest shows the lung has not expanded. Put in drainage-tubes without fenestra, since granulations had crowded into the former tubes so as to nearly occlude them. Sent boy to a farm near Whitewater, Wisconsin, with instructions to keep tubes in position and syringe out the cavity once a day with warm water that had been boiled. Ten weeks later, on August 25, he returned looking altogether different. Was twenty pounds heavier than when first taken sick, and expressed himself as never feeling better in his life. The tubes had remained in place until a few days before, when they had been crowded out and could not be reintroduced. Physical examination showed the lung expanded, with good motion of the chest and vesicular murmur throughout. In a few days more the opening entirely healed, and the boy left for Norway, perfectly recovered, six months from the commencement of his illness, and four months' wearing of the tube.

It is only within a few years that surgeons have been bold enough to make a section of a rib for empyema. It is, perhaps, but natural that the operation should be condemned by those who have always used the trocar or incision. To oppose the operation on purely theoretical grounds, and conclude that it is a grave and serious operation, is, he believes, contrary to the facts in the published cases.

In the article alluded to in the commencement of the paper, we find a table by a London writer of thirty-four cases of empyema, treated surgically, occurring in children of from one to ten years of age. Of the number, seventeen recovered after a short section of one rib had been made. The average time of recovery was seven weeks.

Dr. Garnett reasons that had these cases been treated with the trocar and drainage tube, they would in all probability have recovered equally as well. Here is what he says against exsection: " When we consider the fact that the bony fabric of the chest is still in a condition of progressive development, the rapid and excessive deposit of callus following the solution of continuity of a rib protruding into the cavity of the chest, irritating the lung, the inevitable disparity and loss of symmetry in expansion of the two sides of the thorax, the growing condition of the child, which necessarily adapts itself to the physical results of the traumatic interference, establishing often a permanent deformity, associated at times with more or less spondylitis, and greatly restricted respiratory capacity of the chest, the pouch of periosteum from which the excised piece of rib has been dissected, the protracted healing and long-continued dressing of the open wound, augmenting the chances of septicæmia, and exhausting the vital energies and recuperative resources of the little sufferer, we should hesitate before resorting to so grave a surgical procedure, when a more simple and conservative one can be safely and successfully practiced."

Dr. Strong thinks such conclusions should not go unchallenged. One does not find them from a careful study of these cases. He asks: " Is subperiosteal cutting away of a short segment of one rib, with modern surgical treatment, liable to be followed by such grave consequences as the writer referred to would have us to believe? Free drainage in abscess-cavities is the imperative rule at the present day. This is best obtained by making a patulous opening larger than it is possible to make between the ribs. In the first case here reported, the rib has completely reformed. In the second it is growing again. In neither case did callus injure the lung. In both cases lung-expansion and chest-motion and shape are perfect. Septicæmia, present in both cases, was quickly cut short by free irrigation, which is only possible through a large opening."

He would therefore conclude that in these two cases, section of the rib at least added nothing to the gravity of either case.

Prof. E. Fletcher Ingals said, some four years ago he had reported fourteen cases of empyema which he had treated. Since that time he had treated a number of others. He had no experience with the method adopted by the reader of the paper, but one of his patients unfortunately had had the operation done upon him. He does not look with favor on the operation excepting as a last resort to allow of greater contraction of the chest. The author of the paper performed the operation skillfully and his patients had recovered. But he says he can open the chest otherwise with safety to the patient and satisfaction to himself. He had treated several cases of empyema similar to those just reported—one, a very young child, perhaps a year and a half old, where recovery followed simple aspiration. He recalled two others which were similar to the first case reported, in which the disease had run from three to five weeks. Drainage-tubes were inserted through a canula, and the cavity washed out frequently, and in both the drainage-tubes were required only between one and two weeks, and the patients were well within three weeks. He thought they recovered more speedily than they would have done if the rib had been resected. In another case, the patient had been sick longer, two or three months, when first seen by him, and it was perhaps six weeks before the child recovered. In all of these cases the expansion of the lungs and chest-wall became perfect.

Children are quite as likely to recover by this method as when ribs are resected, and we avoid a great danger. He believes that he has seen cases die from free opening in the chest-cavity, and he does not think it wise practice to open the pleural sac in this manner.

Dr. John D. Skeer had not had much experience in exsection of the ribs for drainage purposes, but thought that as far as the operation is concerned it is certainly not a very formidable one when preformed as Dr. Strong had described it, by making the operation subperiosteal. Dr. Skeer had had a great deal of trouble in the drainage of chest-cavities with small drainage-tubes. He recalled cases which he had treated in the army, and he thought now if he had those cases under his care at present that he would exsect a portion of the rib. Rapid flow of matter under these circumstances does not necessarily follow. The opening in the cavity may be small or large, according to the size of the drainage-tubes, and the flow of matter can be moderated according to the requirements of each individual case. The importance of this grows out of the fact that the lung becomes bound down by bands and adhesions which prevent its rapid expansion, and there may possibly be ulcerated spots admitting air into the pleural cavity, and pus into the lung tissues, from too ready expansion of the lung, and it takes time for these bands and adhesions to soften down, relax and expand to accommodate the lung in its natural expansion. We should differentiate between *serous effusion* into the pleural cavity and suppurative inflammation of the pleura.

Dr. H. T. Patrick said his experience with the operation of resection is limited to one case which he had under observation three years ago. It was not a case of simple empyema, but of pneumo-pyothorax, in a boy 10 years of age, of over a year's standing, with considerable retraction of the chest, and a small external opening Three ribs were resected, the sixth, seventh and eighth. The ribs were markedly overlapping one another, and for that reason more was removed. The boy made very satisfactory and rapid recovery. He had abnormal temperature, and was much emaciated, but immediately after the operation, the chest being washed out once a day, he lost his abnormal temperature, and at once began to gain in flesh, and in a few weeks was entirely well. He thinks resection should be limited to this class of cases. He would say that in empyema aspiration, incision and drainage, and resection of the rib should be the order or procedure. In Dr. Strong's second case, the lung's failing to expand well points out one of the weak points of the operation as a primary procedure. Had aspiration been performed first, the conditions would probably have been more favorable for expansion than by making as free an opening as for resection and allowing air to enter the cavity. In aspiration, we follow right in the line of nature's efforts in these cases of retraction of the chest-wall; we allow the chest to fall in and approximate itself to the other surface. There are other dangers connected with this operation as a primary procedure: one is, that there are cases on record where, under an anæsthetic, the cavity has ruptured into a bronchus, and the patient has choked to death. Deaths have also been recorded from simply washing out the cavity.

Dr. N. H. Pierce: "We resect a rib to simply enlarge our area of drainage." In a case that he had in the last month, where he had drawn off nearly three gallons of fluid in three tappings, he had first inserted a drainage-tube of half inch in diameter; this drain was constantly being plugged up by coagula, or whatever it might have been, thus causing retention and absorption of the purulent matter, followed by the usual symptoms. He resected a rib after that, and introduced a drainage-tube one inch in diameter, with the advantage of not having it clogged up, and the patient is making a good recovery. Another point: there have been a number of cases reported from the Berlin clinic of death from drowning, occurring from washing out the cavity while the patient was under ether, there being a communication between the pleural cavity and the lungs. It is well, therefore, that the primary cleansing be delayed until the day following the operation. In three cases of this character, he had let out the entire contents of the cavity, amounting in each case to three or four pints, with no abnormal result, using as an antiseptic lavage a saturated solution of boracic acid. There have been deaths reported from the use in the pleural cavity of carbolic acid, thymol and salycilic acid, and the only agent that has not been proven more or less dangerous when used in the pleural cavity is boracic acid, and Dr. Fraenkle insists that this is the only thing that should be used in this capacity.

Dr. Strong said he was very glad to find that his article had excited so much interest. It is certainly a practical subject, and one we are likely to meet with any day, and he is yet in favor of resection. He gave it as his impression that the first resection was made about fifteen years ago. A point against resection was raised by Professor Ingals—that of the too rapid evacuation of the matter. That point was answered by Dr. Skeer, who said that when we have free evacuation we have a corresponding influx of air which takes the place of pus. He believes that to be so. In the two cases reported there was considerable cough at the time the chest was opened, but that quickly subsided. He had no fear that there would be rupture of the lung; he could feel the pleura three-fourths of an inch thick. Until some better reasons are given for not operating, he will be in favor of making a free opening by resection—making an opening large enough that one can get into the cavity and evacuate it freely; one cannot get out enough pus through an opening of an eighth of an inch. In the case of the older boy, shreds of lymph as large as the little finger were thrown out for several days. He has no fear in opening the chest freely and draining it freely.

